# Framing Effects on Online Security Behavior

**DOI:** 10.3389/fpsyg.2020.527886

**Published:** 2020-10-21

**Authors:** Nuria Rodríguez-Priego, René van Bavel, José Vila, Pam Briggs

**Affiliations:** ^1^Joint Research Centre, European Commission, Seville, Spain; ^2^Departamento de Análisis Económico: Teoría Económica e Historia Económica, Universidad Autónoma de Madrid, Madrid, Spain; ^3^Center for Research in Social and Economic Behavior (ERI-CES), Intelligent Data Analysis Laboratory (IDAL), University of Valencia, Valencia, Spain; ^4^Department of Psychology, School of Life Sciences, Northumbria University, Newcastle upon Tyne, United Kingdom

**Keywords:** cyber security, gain vs. loss frame, prospect theory, lab experiment, online behavior, nudge, threat assessment

## Abstract

We conducted an incentivized lab experiment examining the effect of gain vs. loss-framed warning messages on online security behavior. We measured the probability of suffering a cyberattack during the experiment as the result of five specific security behaviors: choosing a safe connection, providing minimum information during the sign-up process, choosing a strong password, choosing a trusted vendor, and logging-out. A loss-framed message led to more secure behavior during the experiment. The experiment also measured the effect of trusting beliefs and cybersecurity knowledge. Trusting beliefs had a negative effect on security behavior, while cybersecurity knowledge had a positive effect.

## Introduction

One of the many benefits of the digital transformation of markets is the ability for consumers to access a wide variety of stores and products from any device that connects to the Internet. However, this implies a growth in the complexity of consumer vulnerabilities, often exceeding regulatory efforts (Kucuk, 2016). Chief among these is cybercrime, a growing trend. The proportion of malicious URLs increased from 1 in 20 in 2016 to 1 in 13 in 2017 (SYMANTEC, 2018). In addition, threats in the use of mobile technology increased by 54 percent in 2017, compared to 2016, probably due to the rising use of these devices to access the Internet.

In order to remain secure online, consumers need to preserve their data confidentiality and integrity. They have to make cybersecurity decisions, respond to security-related messages and make adjustments to security-related settings that are not always easily understood (Payne and Edwards, 2008). Many consumers display limited cybersecurity knowledge and skills, despite having daily access to the Internet (Bennett et al., 2008; Bennett and Maton, 2010). Few are fully aware of the consequences of their online behavior, few see their behavior as risky and many fail to follow the recommendations and advice on safety given to them. All of which means that people end up behaving unsafely online, making them vulnerable to cyberattacks.

Such behavioral vulnerability means that people are often the weakest link in the cybersecurity chain (Sasse et al., 2001), which makes them a target. In 2017, 41% of ransomware attacks were against consumers (SYMANTEC, 2018); therefore, a better understanding of users’ security behavior is relevant to tackling the problem of cybersecurity (Yan et al., 2018).

There are many actions consumers could take to increase their online security, including: running and updating antivirus software; using firewalls; not trusting in odd emails from unknown sources (Anderson and Agarwal, 2010); using strong passwords; logging out from sites; using trusted and secure connections, sites and services; providing the minimum amount of personal information needed; and being aware of physical surroundings (Coventry et al., 2014). Yet campaigns and training initiatives aimed at promoting such behaviors are often unsuccessful (Bada et al., 2019) and people generally ignore warnings (Junger et al., 2017), so more is being done to see how behavioral “nudges” might be designed to improve secure behavior and decision-making more directly.

To date a significant body of research has addressed behavioral issues in cybersecurity. For example, recent studies have shown that message framing can affect online shopping decisions (Cheng et al., 2014; Jin et al., 2017) and that privacy priming and security framing can generate safer decision-making around app selection (Chong et al., 2018) or change security incident reporting (Briggs et al., 2017). However, a significant issue with much of this previous research is that it has focused on *perceptions* of privacy and security risks (Miyazaki and Fernandez, 2001) or has over-relied upon *self-reported* past behaviors (Milne et al., 2009), or stated *behavioral intentions* (Anderson and Agarwal, 2010). This paper goes a step further and measures *observed behavior*. This is important, as studies of observed behavior drawn from both psychology and behavioral economics show human decision-making to be both flawed and biased. In part, this is because people are economic in their thinking and avoid processing details explicitly in order to make greater use of their automatic thinking and intuition (Milkman et al., 2009). By investigating actual consumer behaviors, we can understand more about the way such biases impact cybersecurity decision-making.

The present study contributes to a larger research initiative exploring the potential of behavioral insights to improving security behavior. It tests the effectiveness of two similar warning messages, designed to encourage consumers to behave more securely while shopping online, on a range of cybersecurity behaviors. In order to measure these behaviors, we created a lab environment designed to mimic the online shopping experience and provided them with a financial endowment to spend. We then gave participants either a message that focused on the positive outcomes resulting from behaving securely (i.e., a message that framed their behavior in terms of financial gain) or a message focused on negative outcomes resulting from not behaving securely (i.e., a message that framed their behavior in terms of financial loss). Critically, our messages reflected an actual financial gain or loss to the consumer. This is important to avoid adverse effects generated by giving supplemental warning messages that are not properly integrated into the task (Junger et al., 2017).

The rest of this article is structured as follows: section “Literature and Hypotheses” presents the literature review on framing effects and the hypotheses. Section “Materials and Methods” describes the methodology and the experimental procedure; section “Results” presents the results; and section “Conclusion” offers some conclusions.

## Literature and Hypotheses

Individuals will react differently depending on how information is presented to them. In particular, when asked to choose between two options with the same expected value, people will be influenced by whether the outcome is framed as a gain (e.g., likelihood of winning) or as a loss (e.g., likelihood of losing). The frame does not alter the communicated content – it just presents it differently (Tversky and Kahneman, 1981; Druckman, 2001).

In their seminal work, Tversky and Kahneman (1981) presented experimental subjects with two options. One offered a certain outcome and the other offers an uncertain (i.e., risky) outcome. Both options had the same expected value (i.e., utility *x* probability). Options were framed in terms of gains or in terms of losses. Subjects tended to prefer the option of a certain (i.e., non-risky) gain over a risky gain. Conversely, they preferred options with an uncertain (i.e., risky) loss over a certain loss. In other words, people tend to avoid risks when facing the prospect of gains, but will seek risks to avoid prospective losses.

Loss aversion, or negativity bias, suggests people assign stronger values to negative feelings than to positive ones (Kahneman and Tversky, 1979; Rothman and Salovey, 1997). The impact and sensitivity of negative information, therefore, will be higher (Cacioppo et al., 1997; Baumann et al., 2019). For example, individuals display more distress when thinking about losing an amount of money, than the enthusiasm they exhibit for winning the same amount (McGraw et al., 2010). It follows that people will be more motivated to avoid losses than to pursue a gain of equal value (Rozin and Royzman, 2001; Vaish et al., 2008).

When an element of risk is introduced, the framing effect is more nuanced. In particular, in the gain frame, the risky prospect of having some losses is undesirable compared to the certain option of not having any losses. In the loss frame, the certain prospect of having some losses is undesirable compared to a risky prospect which could avoid losses altogether. Hence, in the gain frame people seek certainty and in the loss frame they accept risk (Zhang et al., 2017). In behavior change interventions, therefore, when individuals face a decision that involves a risk of obtaining an unpleasant outcome (e.g., cancer screening), loss-framed messages should be more effective. On the other hand, when the perceived risk of the unpleasant outcome is low, or when the outcome is pleasant (e.g., engaging in physical activity), a gain-framed message should work better (Rothman et al., 2006).

However, what can be expected of gain- and loss-framed messages in behavior change interventions more generally, where the element of risk is not present? The literature is ambiguous in this regard. On the one hand, interventions using a loss frame should be more effective in generating behavior change, simply because “losses loom larger than gains,” as described above (see e.g., Hong et al., 2015). However, a number of sources in the literature argue that gain framing can also be effective as a longer-term intervention. In a meta-analysis of 93 disease prevention studies, gain-framed appeals were more persuasive than loss-framed appeals, although the difference was quite small and attributable to success in gain-framed messages promoting dental hygiene (O’Keefe and Jensen, 2008). Other sources report no significant differences overall, e.g., O’Keefe and Nan (2012) in a meta-analysis of vaccination behavior.

Other factors can mediate subjects’ response to a framed message, such as the level of involvement with the issue, perceived self-efficacy, cultural background, the level of riskiness of the behavior itself, and socio-demographics (Maheswaran and Meyers-Levy, 1990; Banks et al., 1995; Rothman et al., 1999; Millar and Millar, 2000; Meyers-Levy and Maheswaran, 2004; Uskul et al., 2009; Lim and Noh, 2017). For example, in exploring the effects of interventions to reduce alcohol consumption, gain framed messages were more effective with those with low issue involvement, but loss-framed messages were found to be more effective in those with high issue involvement (de Graaf et al., 2015). In our own study, we ensured high issue involvement by making final payoff to the participants contingent upon their cybersecurity behavior and would therefore expect to see some cybersecurity benefits from a loss-framed message.

### The Cybersecurity Context

Translating these findings to the cybersecurity context, we can see that to date, no studies have measured the direct behavioral impacts of a gain or loss framed cybersecurity message, although we can find one study that captures the advice a participant would offer to a fictional friend, following a gain-framed or loss-framed cybersecurity incident. Specifically, Rosoff et al. (2013) conducted a study in which people were presented with a set of scenarios in which they had fictional “prior experience” of a cybersecurity problem and were then asked to “advise a friend” as to the right action to take. Gain and loss framed messages were used to describe the potential outcome of a risky cyber choice with the gain-framed messages endorsing the safe, protective behaviors and the loss-framed messages warning of the consequences of risky action. For example, in a scenario about downloading music, the gain frame explained the actions to take for the friend to avoid the risk of acquiring a virus whereas the loss-frame highlighted the risk of them acquiring a virus. The authors found that the more the focus was on loss, the more likely participants were to make safer cybersecurity decisions. From this limited evidence of loss vs gain framing in the cybersecurity context, then, it would seem that losses do indeed loom larger than gains.

In our experiment, building upon the example above, we assume a loss-framed security message should be more effective in ensuring secure online behavior than a gain-framed message. We can also assume that, as the financial losses are real in our own paradigm, participants have high level of involvement, which would also contribute to loss-framing’s effect. Based on these insights, we postulate the following hypothesis.

*Hypothesis 1:* The group exposed to the loss-framed message will show more secure online behavior than the group exposed to the gain-framed message.

We also consider other factors that could mediate the effect of the interventions tested. Trust is essential in the e-commerce environment as the process of buying online entails some risks, such as sharing personal information with an unknown seller. As a multidimensional construct, it refers to integrity, benevolence and predictability among other factors (McKnight et al., 2002; Gefen et al., 2003). Lack of trust toward an e-commerce seller may prevent users from buying online (Jarvenpaa et al., 1999; Grabner-Kräuter and Kaluscha, 2003; Gefen and Heart, 2006), conversely, trusting the vendor may facilitate online purchasing (McCole et al., 2010). This begs the question as to whether trust can lead to more reckless online behavior. It is an interesting issue and one which suggests an extension of the typical trust relationship in which vendor trust is a gateway to online purchasing. Here we ask whether vendor trust lead to riskier behavior all round. We would expect this to be the case, considering the antecedents of trust as discussed by Patrick et al. (2005), who point out how important trust is as a facilitator of social engineering attacks such as phishing, where familiarity with logos and trade names can lead consumers to erroneously place trust an online message. In this study, we wanted to assess whether trust in an online vendor can similarly create a “trust trap,” effectively inducing a false sense of security that leads to a reduction of cybersecurity behaviors. Hence, we postulate that subjects who are more trusting will behave less securely as they may have confidence on vendor’s goodwill and will not take the necessary steps to protect themselves. We measure *trusting beliefs* combining the scale developed by McKnight et al. (2002) and the one by Jarvenpaa et al. (1999). It provided a high internal consistency (α = 0.93).

*Hypothesis 2:* Participants who exhibit higher levels of trust toward the vendor will show less secure online behavior than participants who exhibit lower levels of trust.

We also included a measure in our model related to *cybersecurity knowledge*, measured by asking our participants to assess a range of security-related behaviors (i.e., providing minimum information, connecting to a trusted site, logging out, etc. – see for example Coventry et al., 2014). We asked participants to rate the behaviors they thought could prevent them from suffering a cyberattack, using a 5-point Likert scale (1 = It won’t reduce my risk at all; 5 = It will reduce my risk extremely). Internal consistency was tested through Cronbach’s alpha and gave a high reliability of the scale (α = 0.90). We expected higher levels of cybersecurity knowledge would lead to more secure behavior, either directly or through increased self-esteem (see e.g., Tang and Baker, 2016). Note that c*ybersecurity knowledge* was only measured in the post-purchase questionnaire to avoid participants being primed with this information during the experiment. We proposed the following hypothesis:

*Hypothesis 3:* Participants with a high level of cybersecurity knowledge will display more secure online behavior than participants with a lower level of knowledge.

## Materials and Methods

### Experimental Procedure

We conducted a laboratory experiment with 120 participants, 60 per treatment^1^. The target population consisted of internet users who had purchased at least a product or a service online in the last 12 months. The participants were selected following a quota design for the sample of both treatments. The quotas were obtained from Eurostat’s Annual Survey of Access and Usage of ICT in Households and Individuals 2013, which established that internet users who purchased a good or service online in the previous 12 months in Spain were 51.7% men and that 40.6% of the Internet users were under 35 years of age. The sample was obtained from the subject pool managed by the laboratory of experimental economics of the ERI-CES (University of Valencia) with more than 25,000 volunteers. The recruitment system of the lab opened a call on its web page, only visible to those participants already registered in the database. Participants had to be actual members of the target population and answered filter questions to confirm this point. They were randomly assigned to experimental treatments until the representative quotas for age and gender were completed in each treatment. After that, no more participants of the age group or gender whose quota had been reached were allowed to register for the experiment. Ethical approval was granted by the Experimental Research Ethics Commission of the ERI-CES. Subjects were invited to the experimental laboratory and randomly assigned to a computer station. At the end of the experimental session, they received an anonymous payment in an enveloped identified only by the number of their station.

During the experiment, participants were asked to make several shopping decisions and were assigned an amount of money (an endowment). The incentive for participating in the experiment was divided in two. They received a fixed show-up fee for participating in the experiment and a variable fee that depended on the decisions they made during the online shopping process and on the random event of suffering a cyberattack. Subjects were told that they could receive a random cyberattack during the experiment. To increase the ecological validity of the experiment and to establish a decision environment similar to real-world Internet use, subjects were informed that the probability of being attacked would depend on the level of security of their online behavior. No specific information on which decisions actually increased or reduced this security level was provided to them. The use of performance-related incentives was relevant in this context to simulate the risks they might take when going online. In the lab, it is not possible to introduce a virus in their computer or make them feel the threat of a cyber-attack, since participants are not using their own computer. Specifically, the fact of suffering the random cyberattack would damage them by reducing their variable payoff at the end of the experiment. Consequently, if they behaved unsafely during the experiment, they could suffer a simulated cyberattack, and they would earn less money. On the contrary, if the behaved safely during the experiment, the probability of suffering a cyberattack would be the lowest and they would receive more money. This mechanism generated an incentive that is aligned with those in real-life situations: subjects aim to reduce the probability of suffering a random cyberattack.

After reading the instructions, and before the shopping experience began, participants filled a questionnaire with sociodemographic items. At the end of the purchase process, they completed a second questionnaire. It included questions related to trust in the e-commerce provider and cybersecurity knowledge.

In the experiment, participants had to buy a real product (a desktop wallpaper). They also had to make several security decisions, although – as mentioned earlier – they were not explicitly told about the potential consequences of each of these decisions. The intention was to let them behave as they would do in a non-experimental environment, where no feedback on security performance is available.

At the end of the experiment, participants had to answer a second questionnaire. After this post-experimental questionnaire, we provided participants with information on their accumulated probability of suffering a cyberattack due to their navigation. A random process then determined if they suffered the cyberattack or not (based on the above-mentioned probabilities). If they suffered the cyberattack, they would lose part of their variable endowment.

### Experimental Conditions

We assigned participants to one of two experimental conditions showing different security messages. The experimental conditions presented a message focusing on the possible positive (i.e., gain-framed) and negative (i.e., loss-framed) outcomes related to their security behavior. Before they had to make any security-related decision, a message appeared as a pop-up in the center of the screen. Participants had to close the pop-up window to continue with the experiment. Then, the message moved to the upper part of the screen. The gain-framed message stated, “*Navigate safely*. *If you do, you could win de maximum final endowment.*” The loss-framed message stated, “*Navigate safely*. *If you don’t, you could lose part of your final endowment.*”

### The Dependent Variables

#### Probability of Suffering a Cyberattack

The first behavioral outcome measure in this study, taken from van Bavel et al. (2019), was the probability of suffering a cyberattack at the end of the experiment, which would reduce participants’ variable payment. The probability was in the range of 5 to 65% and was calculated as a product of the five security decisions made during the experiment. From this minimum value of five percent, the selection of an unsecured connection, a non-trusted vendor or not logging out added 12 percentage points each to the probability of suffering a cyberattack. The sign-up process added another 24 percentage points in total. Lack of strength in the selected password added anywhere from zero percentage points (if the password met all seven six security criteria) to 12 points (if it met none). The non-compulsory information provided added between zero (if none of the items were answered) to 12 points (if subjects answered provided all of the items).

The probability of suffering the attack worked as an effective outcome measure of the security level of decisions made by the subjects: if they always proceeded in the most secure way this probability was kept at its minimum value (5%). On the other hand, if they selected the riskiest option at each step of the experiment, the probability reached its maximum value (65%). The maximum probability was set at a higher value than what could be expected when navigating well-known e-commerce sites in the real world. This was done to maintain a wide range of variation in the outcome measure. In addition, since participants did not actually know this value, it had no impact on their online behavior. Finally, although the probability of suffering a cyberattack was not related to the actual chances of suffering a cyberattack outside the experiment, the decisions that determined the probability were based on good security behavior in the real world (Coventry et al., 2014). This lack of prior information on how this variable is measured provided more ecological validity to the experiment. In real online purchases, consumers do not know in which percentage each of their actions is contributing to an increase in their probability of suffering a cyberattack.

#### Cybersecure Behavior

The second behavioral outcome measure was computed as the mean of the five security-related decisions that participants had to make during the experiment, described in more detail below: choosing a secure connection, choosing a strong password, providing minimum information in the sign-up process, choosing a trusted vendor and logging-out.

The decisions of choosing a secure connection, choosing a trusted vendor and logging-out were binary. The strength of the chosen password depended on seven rules that follow the usual parameters (Keith et al., 2007). Providing minimum information on the sign-up process meant completing as few of the eight optional cells requesting personal information. More information on these decisions is provided in the following subsection. Consequently, the variable *cybersecure_behavior* was computed as in Eq. (1).

(1) $$\text{Cybersecurity}\,\_\,\text{behaviour}=\frac{\text{connection}+\frac{\text{password}}{7}+\frac{\text{sign}-\text{up}}{8}+\text{vendor}+\log-\text{out}}{5}$$

### Security-Related Decisions

During the experiment, participants had to make five security-related decisions, which represented actions that users should take to protect themselves from cyberattacks (Coventry et al., 2014). We focused on decisions related to online purchasing processes that could be tested in an experiment. Participants had to make the decisions sequentially as follows:

#### Decision 1: Choosing a Secure Connection

The first action participants had to make was to connect to the experimental intranet. This was in fact a simulated intranet, with the only aim to examine participants′ security decisions. They had two options: they could choose to connect to the intranet through a secure or an unsecured connection. The secure connection forced the participants to wait 60 s and type a password provided on the screen. The purpose was to force them to make an extra effort if they wanted to behave securely. The next screen displayed a processing bar that charged during the connection process. Below the bar, participants could see a button that allowed them to change to an unsecured but immediate connection if they did not want to wait the entire minute. This possibility would let participants to change their mind, as in the real world.

The unsecured connection was an instant connection to the simulated intranet. Participants did not have to wait – the connection time was 0 s and it did not require any password. However, by choosing this option, participants increased their probability of suffering a cyberattack. The objective was to highlight the often intricate process that behaving safely online entails (as opposed to behaving unsafely). Choosing a secure option reflected the *compliance budget* that users weigh to make a decision (Beautement et al., 2009). The options (secure vs. unsecured) appeared randomly on the left or right-hand side of the screen to avoid location having an effect on participants’ decisions.

After connecting to the intranet, participants could see the e-commerce website. It displayed the mock company name and logo, and a link to the terms and conditions. The link contained information about how the data would be managed, used and stored; the rights of the user; and copyright information. All this information complied with the European Data Protection Directive 95/46/EC. Participants had to accept the terms and conditions during the sign-up process by clicking the button “I agree to the Terms and Conditions”.

The homepage was the gate for the subjects to start choosing products. When a subject clicked on a product, a detailed page for that product opened. On this page, the subject could click on the “buy” button to continue with the shopping process, or could go back to see any other products offered.

#### Decision 2: Choosing a Strong Password

Online consumers can prevent unauthorized individuals to exploit their password by creating a long password (Keith et al., 2007), or combining numbers and special characters with letters.

During the experiment, once subjects decided which product to buy, they had to register by creating a username and a password. We measured the level of password strength according to seven common security parameters, which included a minimum number of characters, lower case characters, upper case characters, numeric digit characters, and special characters, and a Boolean check whether password contained the username or email. Each of the seven criteria would increase the probability of suffering a cyberattack if not met.

#### Decision 3: Providing Minimum Information in the Sign-up Process

During the registration process, after choosing the username and password, participants were asked to provide some personal information. The information required to continue with the process was marked with an asterisk (name, surname, and email), but the remaining information (gender, age, phone number, address, zip code, city, region, and country) was optional. This is the usual kind of information requested in websites, which e-Commerce providers find useful for sending targeted advertising. The secure option was to disclose only the required information. Each of the eight non-compulsory items increased the probability of suffering a cyberattack. While the other four decisions reduced the risk of suffering a cyberattack, this measure went in the opposite direction: the higher the value meant the participant was behaving *less* securely. Therefore, when included in the outcome measure *cybersecure_behavior*, the “sign-up” variable was reversed. Admittedly, this variable had some limitations, as the veracity of the information provided in these non-compulsory items could not be guaranteed. In order to preserve anonymity, the personal data disclosed by participants was not recorded.

From the moment subjects registered until the end of the purchasing process, the top right-hand side of the screen displayed the text “Welcome” followed by their username, next to which was a button to log out of the e-commerce website.

#### Decision 4: Choosing a Trusted Vendor

Once subjects had completed the registration process, they had to select their choice of product (desktop wallpaper) between four possible options. Each of the products displayed a different picture, but the decision of choosing one of them was not relevant for the study, as it did not involve any secure or unsecure option. After that, participants had to choose between two vendors. Both vendors offered the same product, and were randomly ordered. The price offered by the first vendor for the product was zero. In this case, the link to download the product had no security signals (no image for an e-trusted site). The simulated link for this supplier was http (Hypertext Transfer Protocol). The second vendor offered the product for €2, but the link to download it was of the https (Hypertext Transfer Protocol Secure) type and appeared next to an image indicating it was an e-trusted site. Different prices depending on the security of the provider reflected how, in the real world, users can obtain products for free, but possibly compromising their security. If the participants chose the unsecured option (for free), they would increase the probability of suffering a cyberattack.

#### Decision 5: Logging Out

Once subjects had completed the purchasing process, a new screen displayed information about the cost of the purchased product and the amount remaining on their credit cards. A new button indicating “Next questionnaire” appeared at the bottom right-hand side of this screen. However, the secure option was to log out before continuing to the next questionnaire. Participants were not told explicitly to log out, although they were asked to exit the e-commerce website and complete the next questionnaire. If they did not log out, their probability of suffering a cyberattack at the end of the experiment increased.

## Results

In this section, we present the socio-demographic profile of participants in the sample and the ANCOVA model that tested the effect of the treatments, trust beliefs and knowledge on the probability of suffering a cyberattack.

### Sociodemographic Information of the Sample

Quotas were applied by sex and age. Their value was fixed according to the profile of the internet users provided by the Annual Survey of Access and Usage of ICT in Households and Individuals in 2013, where 51.7% of Internet users were men and that 40.6% of the Internet users were under 35 years of age. Age ranged between 19 and 69 years. Sixty percent of participants were older than 32 and the mean age was 36.9 years. We provide further sociodemographic information on the educational level and employment status of the participants in Table 1.

**TABLE 1:** Sociodemographic characteristics of participants^1^.

***Education level***	**%**
No studies	0.83
Primary or lower secondary education	5.00
Upper secondary education and post-secondary, non-tertiary education	54.17
Bachelor’s degree or equivalent	31.67
Postgraduate degree	4.17
PhD	4.17

***Employment status***	**%**

Self-employed	3.33
Employed by a public or private institution	33.33
Unemployed	24.17
Homemaker	1.67
Student	35.00
Disabled	0.00
Retired	2.50

*^1^This table provides information on education level and employment status of the sample. Further information on gender and age is included in the subsection Sociodemographic Information of the Sample.*

### Main Effects on the Probability of Suffering a Cyberattack

**FIGURE 1:**
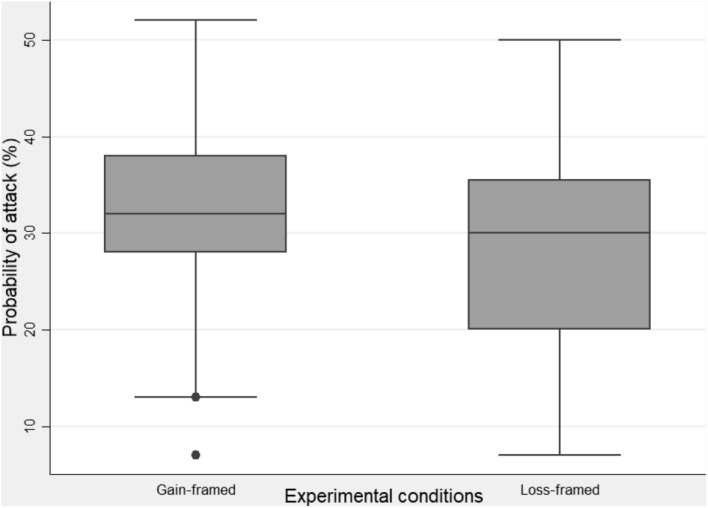
Box-plot of the probability of suffering a cyberattack by experimental group.

The mean probability of suffering a cyberattack during the experiment was higher in the gain-framed treatment (*M* = 33.16, SD = 10.04) than in the loss-framed treatment (*M* = 28.43, SD = 11.74; Figure 1). A two-tailed *t*-test comparing the means of the probability of suffering a cyberattack between the two treatments (gain vs. loss) showed a significant effect [*t*(188) = 2.37, *p* = 0.019]. A *post hoc* analysis using jStat with an alpha of 0.05 gave a power of 0.636. A loss-framed message appeared to be more effective in generating secure behavior, lending some support to Hypothesis 1.

We estimated a first regression model taking as dependent variable the probability of suffering a cyberattack. The explanatory variables were: (i) the treatments; (ii) cybersecurity knowledge, trusting beliefs; and (iii) the interactions between the treatments and the other explanatory variables. This first model showed no significant results for the interactions between the treatments and the other independent variables. In other words, the effect of the gain vs. loss-framed messages did not depend on cybersecurity knowledge or trusting beliefs.

Table 2 provides the estimation of the final model. It shows that the loss-framed message significantly decreased the probability of cyberattack compared to the gain-framed message [*t*(116) = −2.36, *p*-value = 0.020]. The estimated values of the coefficients show that a loss-framed message reduces the probability of suffering a cyberattack by 4.61%. This result confirms support for Hypothesis 1.

**TABLE 2:** Estimated coefficients of the final model for the probability of suffering a cyberattack.

	**Estimate**	**Std. Error**	***t*-value**	**Pr(>|t|)**
Loss-framed^1^	–4.61	1.95	–2.36	0.020
Knowledge^2^	–3.41	1.60	–2.13	0.036
Trusting beliefs^3^	2.92	1.36	2.15	0.034
Cons	–35.83	6.74	5.32	0.000

*^1^The gain-framed condition was taken as baseline for the data analysis.*

*^2^The variable *Knowledge* was estimated as a mean of the values obtained in each of the 10 items that comprised the Knowledge Scale. This scale is provided in the Supplementary Table A2. Each of the items were measured in a 5-point Likert scale.*

*^3^The variable *Trusting beliefs* was estimated as a mean of the values obtained in each of the 10 items that comprised the Trusting Beliefs Scale. This scale is provided in the Supplementary Table A1. Each of the items were measured in a 5-point Likert scale.*

Second, *trusting beliefs* had a significant effect on the dependent variable [*t*(116) = 2.15, *p*-value = 0.034]. Participants who placed higher levels of trust in the vendor showed less secure behavior during the experiment. Hypothesis 2 is also supported.

Finally, knowledge of cybersecurity risks affected the probability of suffering a cyberattack in an inverse relationship (more knowledge meant less likelihood of an attack) [*t*(116) = −2.13, *p* = 0.036]. Hypothesis 3 is also supported.

Tables 3–7 show participants’ behavior in each of the five decisions they had to make during the experiment, by experimental treatment. Regarding the first behavior (Table 3), all subjects decided to choose a secure connection over the unsecured one, no matter the framing of the message. Perhaps, at this early stage of the process, all subjects were concerned with navigating securely, as they had just read the security message that appeared in the center of the screen. After closing the pop-up, the message would only appear in the upper part of the screen during the rest of the experiment.

**TABLE 3:** Decision 1 – choosing a secure connection by treatment^1^.

**Treatment**	**Connection security**		
	**Unsecured**	**Secure**	**Total**
Gain-framed^2^	0	60	60
%	0	100.00	100.00
Loss-framed^2^	0	60	60
%^3^	0	100.00	100.00
Total	0	120	120

*^1^Decision 1 was binary. It takes the value of 1 for choosing a secure connection, and 0 for choosing an unsecure connection.*

*^2^Values for gain-framed and loss-framed are given in absolute terms.*

**TABLE 4:** Decision 2 – choosing a strong password by treatment^1^.

**Treatment**	**Password strength [1–7]**							
	**1**	**2**	**3**	**4**	**5**	**6**	**7**	**Total**
Gain-framed^2^	1	2	16	17	23	1	0	60
%	1.67	3.33	26.67	28.33	38.33	1.67	0.00	100.00
Loss-framed^2^	0	0	16	20	17	6	1	60
%	0.00	0.00	26.67	33.33	28.33	10.00	1.67	100.00
Total	1	2	32	37	40	7	1	120

*χ^2^(6, *N* = 120) = 8.7147 Pr = 0.190.*

*^1^Values for decision 2 ranged between 0 and 7 depending on the number of criteria that participants met for password strength. All of the subjects met at least 1 criteria.*

*^2^ Values for gain-framed and loss-framed are given in absolute terms.*

**TABLE 5:** Decision 3 – providing minimum information in the sign-up by treatment^1^.

**Treatment**	**Information provided in the sign-up [1–8]**									
	**0**	**1**	**2**	**3**	**4**	**5**	**6**	**7**	**8**	**Total**
Gain-framed^2^	4	1	5	2	0	1	5	3	39	60
%	6.67	1.67	8.33	3.33	0.00	1.67	8.33	5.00	65.00	100.00
Loss-framed^2^	7	3	6	1	2	1	0	4	36	60
%	11.67	5.00	10.00	1.67	3.33	1.67	0.00	6.67	60.00	100.00
Total	11	4	3	2	2	2	5	7	75	120

*χ^2^ (8, *N* = 120) = 9.5053 Pr = 0.301.*

*^1^Values for decision 3 ranged between 0 and 8 depending on the number of non-compulsory cells that participants filled in when registering in the e-commerce website.*

*^2^Values for gain-framed and loss-framed are given in absolute terms.*

**TABLE 6:** Decision 4 – choosing a trusted vendor by treatment^1^.

**Treatment**	**Trusted vendor**		
	**Untrusted**	**Trusted**	**Total**
Gain-framed^2^	18	42	60
%	30.00	70.00	100.00
Loss-framed^2^	10	50	60
%	16.67	83.33	100.00
Total	28	92	120

*χ^2^ (1, *N* = 120) = 2.981, *p* = 0.084.*

*^1^Decision 4 was binary. It takes the value of 1 for choosing a trusted vendor, and 0 for choosing an untrusted vendor.*

*^2^Values for gain-framed and loss-framed are given in absolute terms.*

**TABLE 7:** Decision 5 – logging out by treatment^1^.

**Treatment**	**Logging out**		
	**Stay connected**	**Log out**	**Total**
Gain-framed^2^	48	12	60
%	80.00	20.00	100.00
Loss-framed^2^	39	21	60
%	65.00	35.00	100.00
Total	87	33	120

*χ^2^ (1, *N* = 120) = 3.3856 Pr = 0.066*

*^1^Decision 5 was binary. It takes the value of 1 for logging-out after the purchase, and 0 for staying connected.*

*^2^Values for gain-framed and loss-framed are given in absolute terms.*

The second decision was to choose a password (Table 4). As mentioned before, password strength was measured according to seven common security parameters. Each of the seven criteria would increase the probability of suffering a cyberattack if not met. Results show that subjects in the loss-framed message condition met at least three of the seven criteria, and one of them met all criteria. In the gain-framed condition, three participants met fewer than three criteria and none of them met the seven criteria.

Table 5 shows the quantity of information that subjects provided during the sign-up process. There were eight non-compulsory items included in the sign-up information. Results show that 6.67% of subjects in the gain-framed condition provided no information apart from the compulsory, compared to 11.67% in the loss-framed condition.

The fourth decision was to choose between a trusted vs. untrusted vendor (Table 6). Here, 30% of participants in the gain-framed treatment decided to choose the untrusted vendor, compared to a 16.67% of subjects who visualized the loss-framed message.

The last decision was to log-out or stay connected at the end of the purchase process (Table 7). The amount of participants who chose the secure option (i.e., logging-out) was a 15% higher in the loss-framed condition than in the gain-framed one. Finally, although we found differences between both treatments in some of the individual security-related decisions, none of them was statistically significant.

### Main Effects on Cybersecure Behavior

Table 8 provides the estimated coefficients of the model for the dependent variable *cybersecure_behavior*. It shows that the loss-framed message significantly increased cybersecure compared to the gain-framed message [*t*(116) = 2.46, *p*-value = 0.015]. A *post hoc* analysis using jStat with an alpha of 0.05 gave a power of 0.653. The estimated values of the coefficients show that a loss-framed message increases cybersecure behavior, which supports Hypothesis 1.

**TABLE 8:** Estimated coefficients of the final model for cybersecure behavior.

	**Estimate**	**Std. error**	***t*-value**	**Pr(>|t|)**
Loss-framed^1^	0.07	0.03	2.46	0.015
Knowledge^2^	0.05	0.03	2.16	0.033
Trusting beliefs^3^	–0.05	0.02	–2.24	0.027
Cons	0.50	0.11	4.59	0.000

*^1^The gain-framed condition was taken as baseline for the data analysis.*

*^2^The variable *Knowledge* was estimated as a mean of the values obtained in each of the 10 items that comprised the Knowledge Scale. This scale is provided in the Supplementary Table A2. Each of the items were measured in a 5-point Likert scale.*

*^3^The variable *Trusting beliefs* was estimated as a mean of the values obtained in each of the 10 items that comprised the Trusting Beliefs Scale. This scale is provided in the Supplementary Table A1. Each of the items were measured in a 5-point Likert scale.*

*Trusting beliefs* had also a significant effect on the dependent variable [*t*(116) = −2.24, *p*-value = 0.027], which confirms Hypothesis 2. Participants who placed higher levels of trust in the vendor showed less secure behavior during the experiment.

Third, *knowledge* of cybersecurity risks influenced positively cybersecure behavior, providing support for Hypothesis 3 [*t*(116) = 2.16, *p*-value = 0.033].

## Conclusion

In this research, we examined the effect of security messages on Internet users’ behavior during an online shopping process. Our first hypothesis was that, compared to gain-framed messages, loss-framed messages would be more effective in ensuring participants behaved securely during this process. The findings support this hypothesis.

This paper then makes a contribution by extending work on loss aversion bias, where individuals assign stronger values to negative feelings than to positive ones (Kahneman and Tversky, 1979; Rozin and Royzman, 2001; Ert and Erev, 2008; Vaish et al., 2008; McGraw et al., 2010), and shows its relevance to the cybersecurity context.

A number of recent studies, including Junger et al. (2017), suggest the presence of threat information can backfire if it takes the form of a general warning, yet in our study threat or loss information was effective. Two aspects of our loss-framing might be relevant here.

Firstly, our loss message was tied explicitly to a financial loss outcome (i.e., it did not simply cite some kind of general threat). This means our result is in line with the idea that messages focused on the negative consequences of non-compliance are more persuasive (Cacioppo et al., 1997) when participants are more involved, i.e., more motivated to change. In our case, participants stood to lose money if they behaved insecurely and so motivation (or involvement) was high (cf. de Graaf et al., 2015). Our findings also demonstrate that the “loss looms larger” message does apply to cybersecurity behavior and is not limited to behavioral intentions [as with the Rosoff et al. (2013) study].

Secondly, our loss message was yoked to a behavioral nudge to navigate safely (i.e., we told consumers what they needed to do to avoid loss). Therefore, our intervention was aligned to recent findings that show that threat (or loss) appeals in isolation fail, but they can be effective when presented in conjunction with coping messages that direct consumer behavior (van Bavel et al., 2019).

With regard to trusting beliefs, subjects who trusted the vendor more performed worse on the experiment, meaning that they made decisions that entailed more security risks, ending with a higher probability of suffering a cyberattack. This result supports our second hypothesis and ties in with the literature on phishing and other forms of social engineering wherein trust in a known vendor is explicitly used to overcome defensive behaviors (Patrick et al., 2005). Consequently, trusting beliefs and their influence on users’ performance as the weakest link in this wider cybersecurity chain is an issue that should be further investigated.

It should not be surprising that trust is an issue in this space. Firstly, we know that trust in an e-commerce vendor not only increases click-through intention, but also decreases malware risk perception (Ogbanufe and Kim, 2018). Secondly, and more importantly, we have seen the “weaponisation” of trust, with the huge rise in cybersecurity attacks that draw on social engineering principles to create an illusion of trust. Consumers are often led to believe that communication is with a “trusted” party, when in fact some imitation of that trusted party occurs (e.g., in phishing attacks). Trust, when exploited in this way, has negative implications for both genuine vendors and consumers and it is interesting to explore the kinds of “nudges” that might make people less willing to trust in a superficially familiar message or website (e.g., Moody et al., 2017; Nicholson et al., 2017).

The results regarding the effect of knowledge about cybersecurity support our third hypothesis. Subjects with a higher level of agreement that the listed security actions would prevent them from being attacked behaved more secure during the experiment. We can extract from this that subjects who have a clear concern of what secure behavior means may perform better when making security decisions – a finding again in keeping with recent work on the role of promoting “coping interventions” as part of cybersecurity protection (e.g., Tsai et al., 2016; Jansen and van Schaik, 2017; van Bavel et al., 2019).

Our findings from the questionnaire confirm that consumers’ trust makes them vulnerable and that knowing what secure behavior is improves security decisions. Based on our experimental findings, however, we would contend that a fear-arousal behavioral component that describes a meaningful loss, but that also describes the way to avoid that loss, could be effective as a cybersecurity intervention.

## Data Availability Statement

The datasets generated for this study can be found in the Mendeley Data Repository (Rodriguez-Priego, Nuria (2020), “Framing effects on online security behavior”, Mendeley Data, V2, doi: 10.17632/sp6cyrfvrv.2).

## Ethics Statement

Ethical approval was granted by the Experimental Research Ethics Commission of the ERI-CES from the University of Valencia. All participants provided informed consent.

## Author Contributions

All authors contributed equally to the work.

## Disclaimer

The views expressed in this article are purely those of the authors and may not in any circumstances be regarded as stating an official position of the European Commission.

## Conflict of Interest

The authors declare that the research was conducted in the absence of any commercial or financial relationships that could be construed as a potential conflict of interest.

## Footnotes

1 This sample was extracted from a larger study with 600 participants testing the effect of different warning messages on security behavior (Rodríguez-Priego and van Bavel, 2016).
