# Supplementary material for: Framing Effects on Online Security Behavior
Source: Front Psychol. 2020 Oct 21;11:527886. doi: 10.3389/fpsyg.2020.527886 (PMC7609889; doi:10.3389/fpsyg.2020.527886)
Supplement: Supplementary file 1 [file Data_Sheet_1.docx]

Supplementary Material

# Supplementary Tables

## Supplementary Table A1: Trusting Beliefs Scale

Please, choose in the table below the level of agreement or disagreement with the statements listed (*Strongly disagree* = 1; *Strongly agree* = 5):

| 1. I believe that “LINEEX Buyvip” would act in my best interest. |
| --- |
| 1. If I required help, “LINEEX Buyvip” would do its best to help me. |
| 1. “LINEEX Buyvip” is interested in my well-being, not just its own. |
| 1. “LINEEX Buyvip” is truthful in its dealings with me. |
| 1. “LINEEX Buyvip” is sincere and genuine. |
| 1. This e-commerce vendor is trustworthy. |
| 1. This e-commerce vendor provides reliable information. |
| 1. This e-commerce vendor keeps promises and commitments. |
| 1. This e-commerce vendor’s behaviour meets my expectations. |
| 1. I find it necessary to be cautious with this store (item reversed in the database). |

## Supplementary Table A2: Knowledge Scale

Which of the following behaviours do you think can help you prevent from being attacked while online? Provide a rating from “*It won´t reduce my risk at all*” (1) to “*It will reduce my risk extremely*” (5).

| 1. Connecting to a trusted connection |
| --- |
| 1. Using a strong password |
| 1. Changing your password frequently |
| 1. Avoid using the same password for different sites |
| 1. Providing minimal information |
| 1. Connecting to a trusted site |
| 1. Logging out |
| 1. Using anti-virus software and firewalls |
| 1. Updating software to the latest version |
| 1. Avoiding access to my personal accounts in public places |

# Supplementary Figures

## Supplementary Figure A1. Intranet connection page with two options


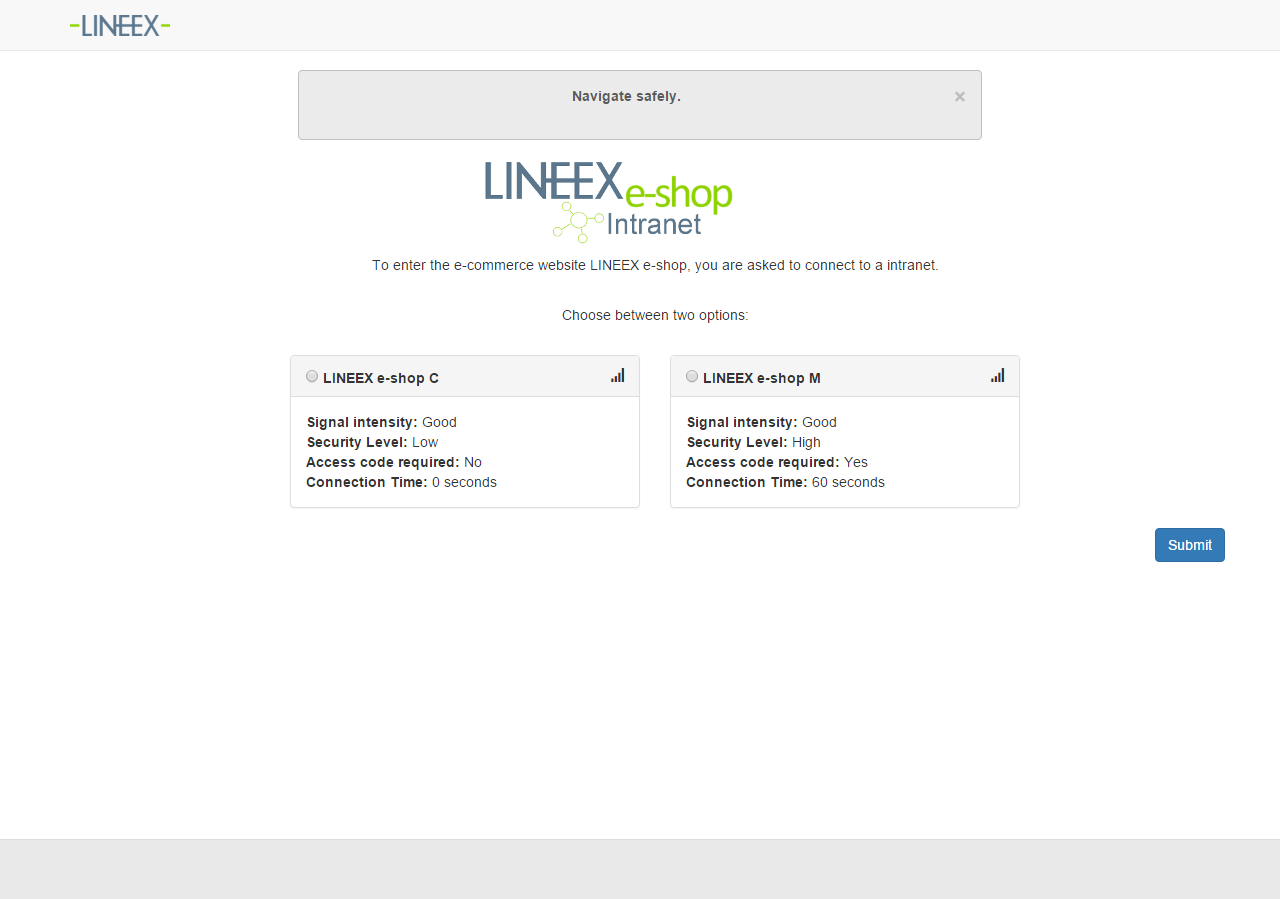


## Supplementary Figure A2: Sign-up and requesting password page


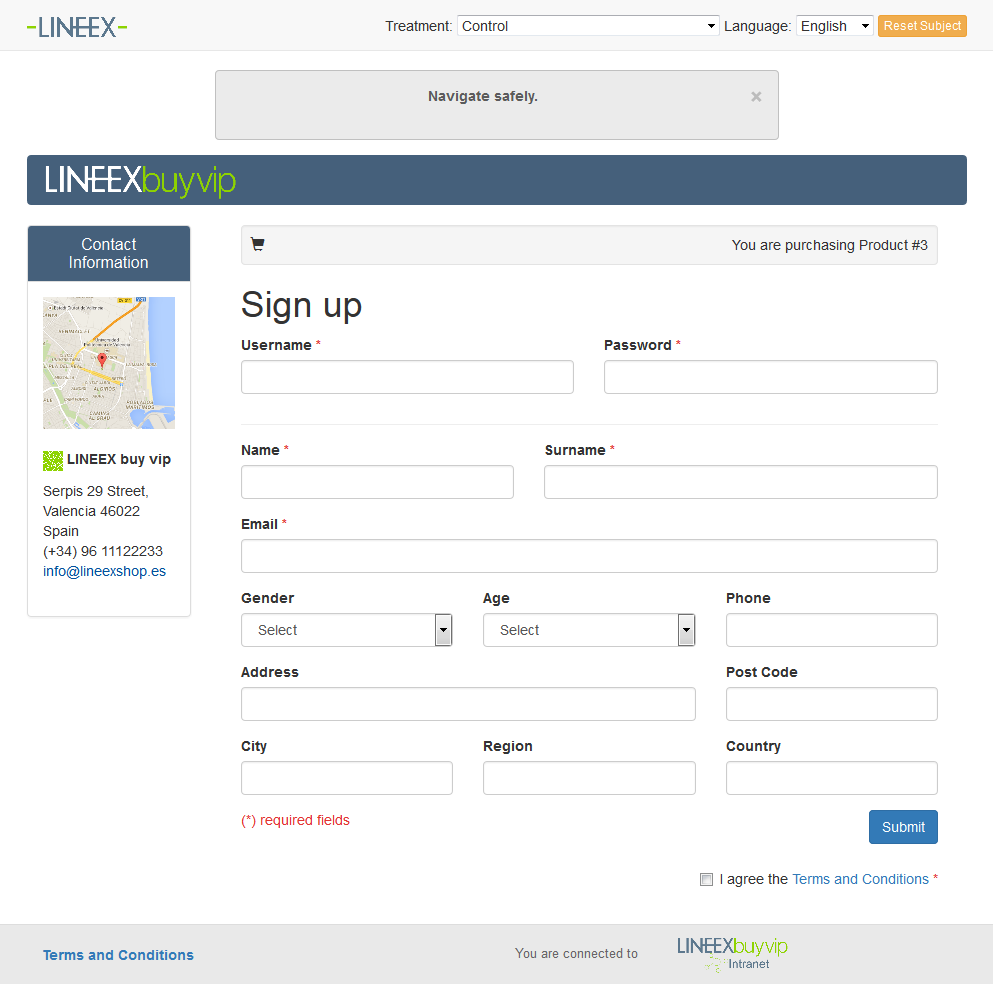


## Supplementary Figure A3: Choice of vendor page


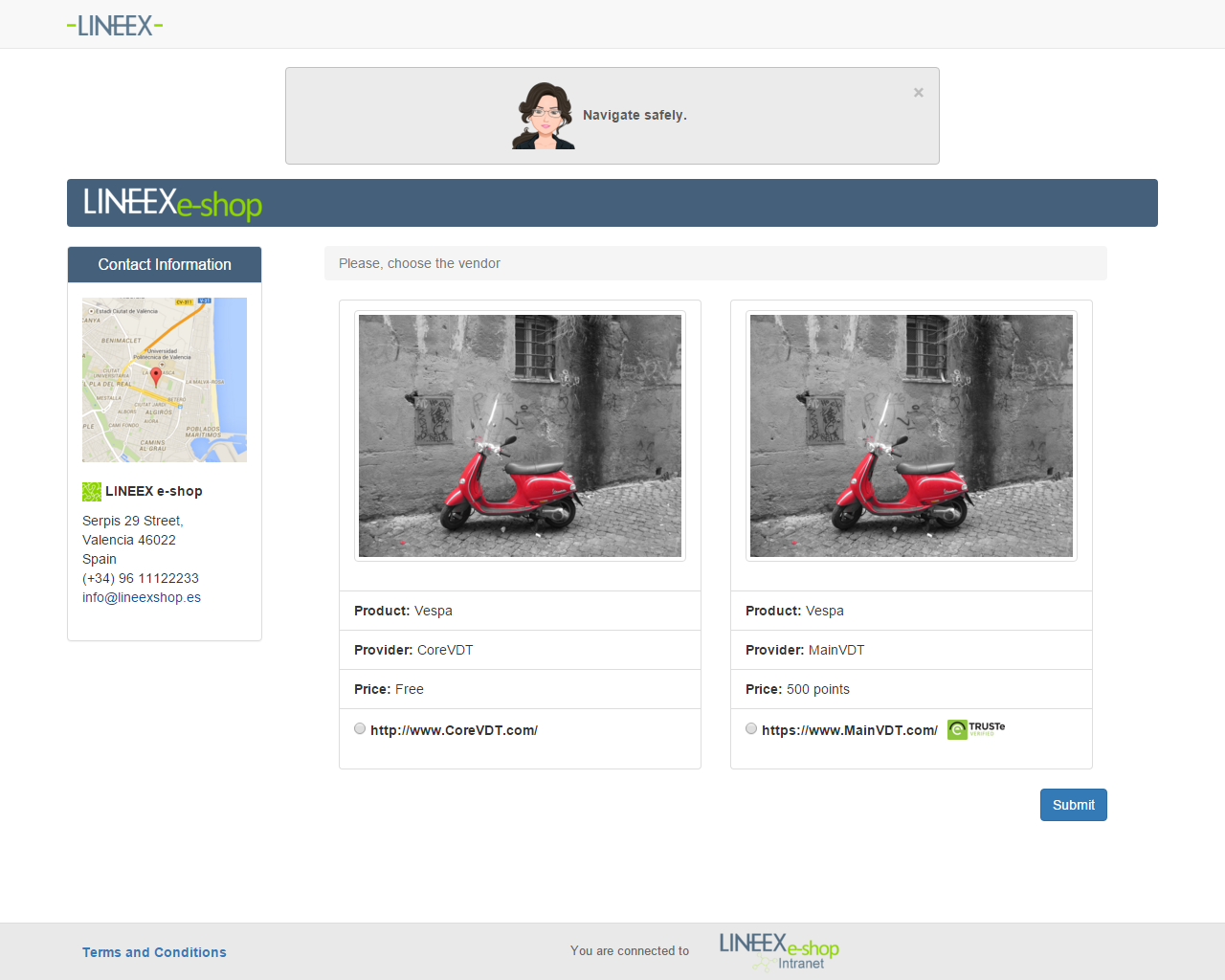


## Supplementary Figure A4: Log-out page


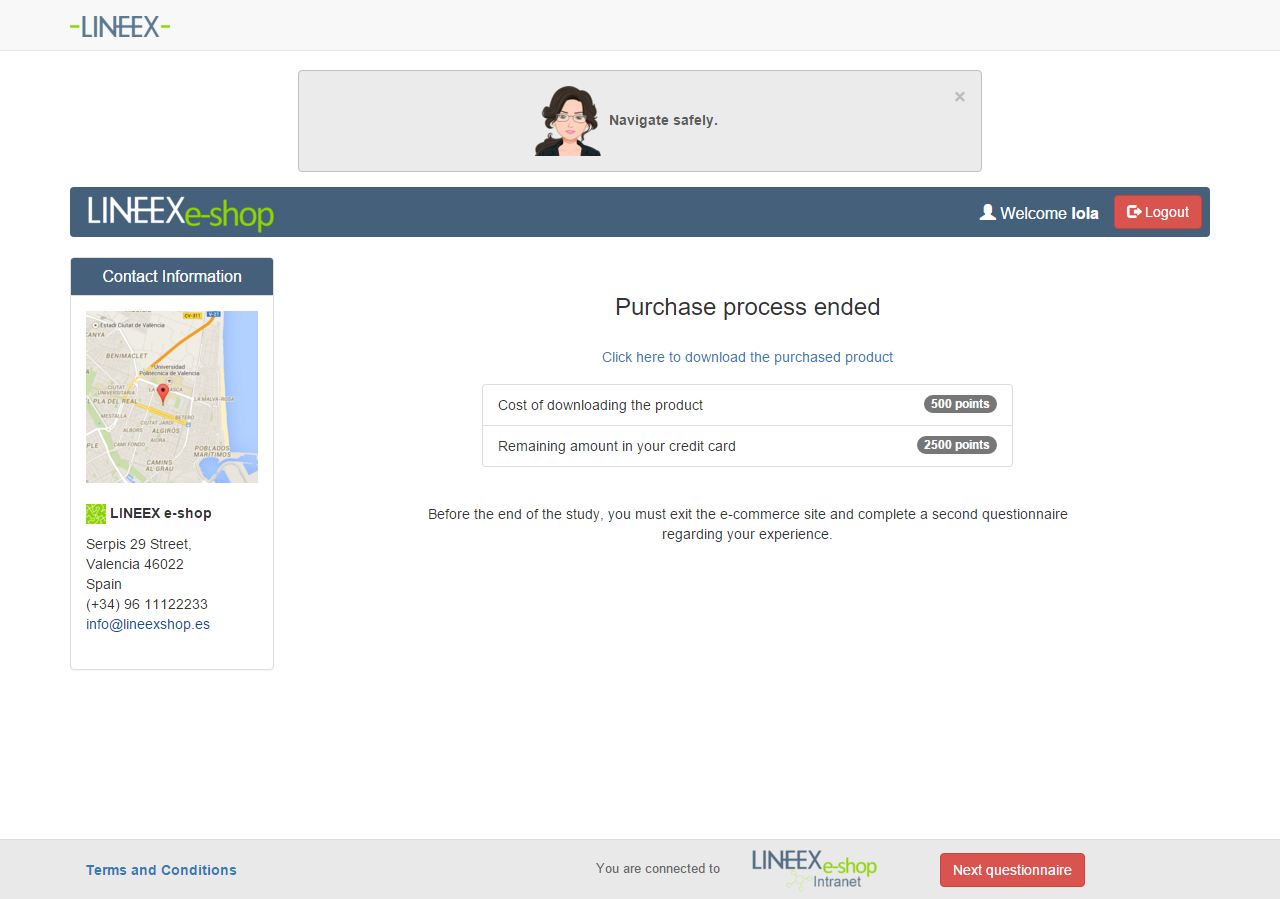


**2.5. Supplementary Figure A5: Probability of suffering a cyberattack**

**
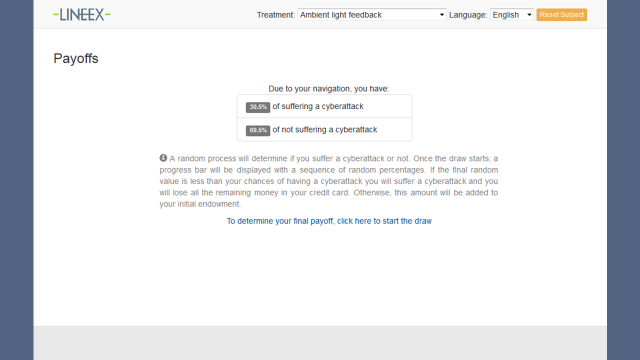
**

**2.6. Supplementary Figure A6: Cyberattack**


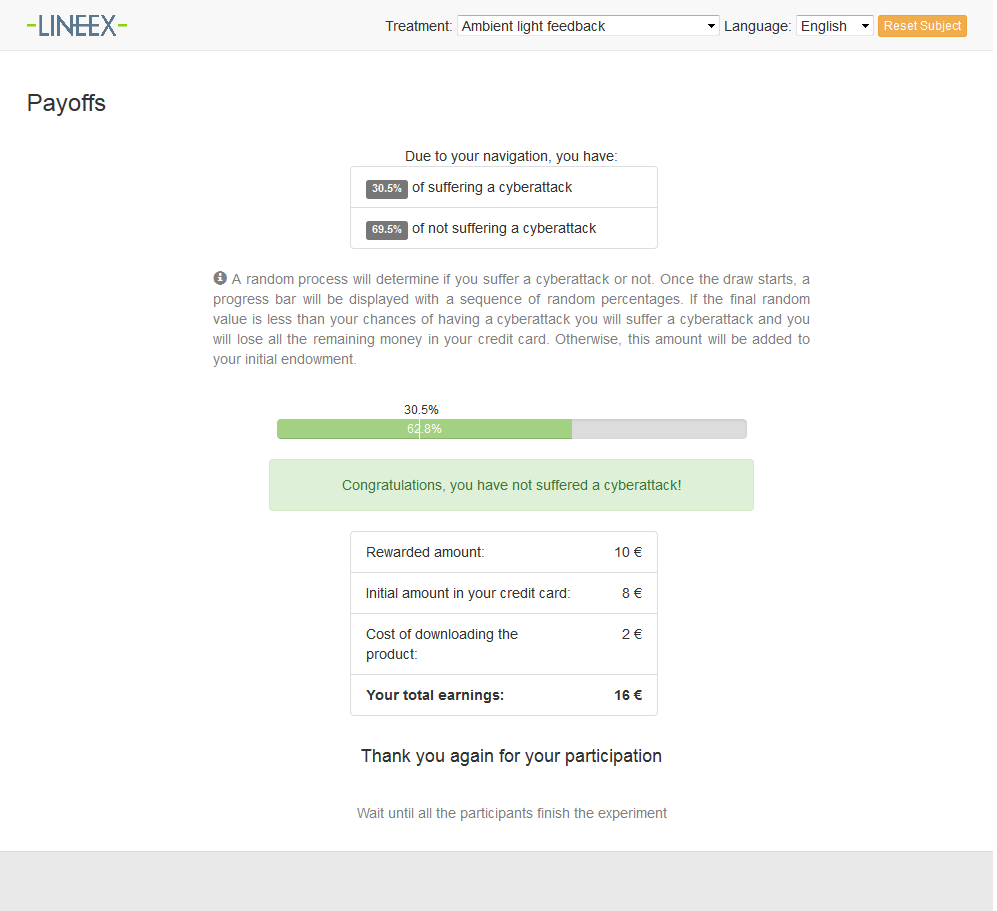


**2.7.** **Supplementary Figure A7: Instructions**

**
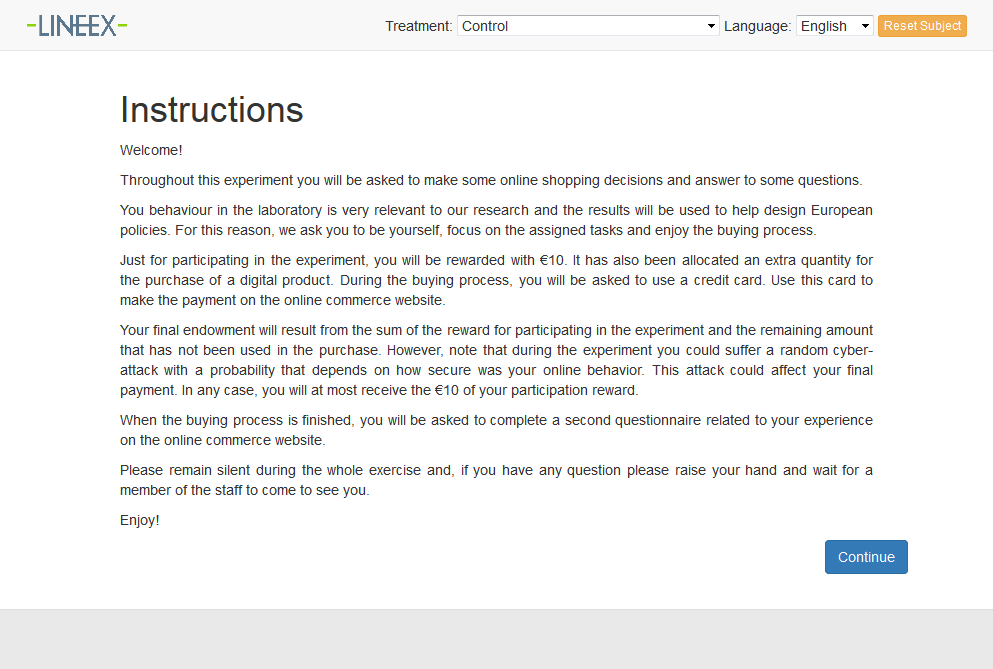
**
